# Machine Learning Approaches Applied to GC-FID Fatty Acid Profiles to Discriminate Wild from Farmed Salmon

**DOI:** 10.3390/foods9111622

**Published:** 2020-11-07

**Authors:** Liliana Grazina, P. J. Rodrigues, Getúlio Igrejas, Maria A. Nunes, Isabel Mafra, Marco Arlorio, M. Beatriz P. P. Oliveira, Joana S. Amaral

**Affiliations:** 1 REQUIMTE-LAQV, Faculdade de Farmácia, Universidade do Porto, Rua de Jorge Viterbo Ferreira, 228, 4050-313 Porto, Portugal; li_grazina@hotmail.com (L.G.); antonianun@gmail.com (M.A.N.); beatoliv@ff.up.pt (M.B.P.P.O.); 2 Research Centre in Digitalization and Intelligent Robotics (CeDRI), Instituto Politécnico de Bragança, Campus de Santa Apolónia, 5300-253 Bragança, Portugal; pjsr@ipb.pt (P.J.R.); igrejas@ipb.pt (G.I.); 3 Dipartimento di Scienze del Farmaco, Food Chemistry Unit, Università del Piemonte Orientale “A. Avogadro”, Largo Donegani 2/3, 28100 Novara, Italy; marco.arlorio@uniupo.it; 4 Centro de Investigação de Montanha (CIMO), Instituto Politécnico de Bragança, Campus de Sta. Apolónia, 5301-857 Bragança, Portugal

**Keywords:** authenticity, fish, *Salmo salar* L., fatty acids, mislabeling, chemometrics, machine learning

## Abstract

In the last decade, there has been an increasing demand for wild-captured fish, which attains higher prices compared to farmed species, thus being prone to mislabeling practices. In this work, fatty acid composition coupled to advanced chemometrics was used to discriminate wild from farmed salmon. The lipids extracted from salmon muscles of different production methods and origins (26 wild from Canada, 25 farmed from Canada, 24 farmed from Chile and 25 farmed from Norway) were analyzed by gas chromatography with flame ionization detector (GC-FID). All the tested chemometric approaches, namely principal components analysis (PCA), t-distributed stochastic neighbor embedding (t-SNE) and seven machine learning classifiers, namely k-nearest neighbors (kNN), decision tree, support vector machine (SVM), random forest, artificial neural networks (ANN), naïve Bayes and AdaBoost, allowed for differentiation between farmed and wild salmons using the 17 features obtained from chemical analysis. PCA did not allow clear distinguishing between salmon geographical origin since farmed samples from Canada and Chile overlapped. Nevertheless, using the 17 features in the models, six out of the seven tested machine learning classifiers allowed a classification accuracy of ≥99%, with ANN, naïve Bayes, random forest, SVM and kNN presenting 100% accuracy on the test dataset. The classification models were also assayed using only the best features selected by a reduction algorithm and the best input features mapped by t-SNE. The classifier kNN provided the best discrimination results because it correctly classified all samples according to production method and origin, ultimately using only the three most important features (16:0, 18:2n6c and 20:3n3 + 20:4n6). In general, the classifiers presented good generalization with the herein proposed approach being simple and presenting the advantage of requiring only common equipment existing in most labs.

## 1. Introduction

In recent decades, the consumption of fish has been increasingly recommended due to its health benefits, mainly related to the prevention of cardiovascular diseases [1]. In particular, fatty fishes from cold waters, such as salmon, are frequently rich in polyunsaturated fatty acids (PUFA), including the essential fatty acids linoleic (18:2n6) and α-linolenic (18:3n3), but also in several omega-3 PUFA such as eicosapentaenoic (EPA, C20:5n3) and docosahexaenoic (DHA, 22:6n3) acids. Besides being components of cell membranes, omega-3 PUFA are involved in the biosynthesis of eicosanoids and have been shown to influence health by affecting cell signaling cascades and gene expression, resulting in decreased expression of inflammatory and atherogenesis-related pathways [2,3]. Moreover, different studies showed that omega-3 PUFA play an important role in altering blood lipid profiles and associate their consumption with improved cardiovascular function and decreased risk of atherosclerosis and peripheral arterial disease [2,3].

In addition to these benefits, fish is largely consumed for its nutritional value and sensory aspects, making it one of the most traded food commodities. In this sense and considering that the world’s wild fish stocks are limited, the production of farmed fish has been steadily increasing in recent last years. In fact, according to the Food and Agriculture Organization (FAO) Globefish Highlights, world fisheries capture was 92.5 million tonnes in 2017 with this figure expected to decrease to 91.3 million tonnes in 2019, by the contrary, fish capture arising from aquaculture is expected to grow from 80.1 to 86.5 million tonnes in the same period [4]. Concerning salmon, from 2000 to 2014, a much stronger increase was verified for aquaculture production (from 898,800 to 2,326,300 tonnes) compared to that of the world’s capture of wild salmon (from 728,000 to 879,000 tonnes) [5]. Aquaculture allows wider consumer access to fish generally at more affordable costs, though it is known that fatty acid composition can significantly vary according to its production method (wild vs. aquaculture). Particularly for salmon, it has been reported that wild salmon generally present higher contents of valuable omega-3 PUFA [6,7,8]. This aspect, together with particular organoleptic characteristics, has driven several consumers to prefer wild salmon. Considering the limited availability of this type of salmon and its growing demand, prices have been increasing significantly, resulting in this product being prone to adulteration by origin mislabelling or even substitution with other lower-cost fish [9,10,11]. Whereas fish species authentication can be performed using well established and straightforward DNA-based methods [12], different approaches have been proposed so far to assess the origin of fish with respect to production method. These include, mainly, the use of nuclear magnetic resonance (NMR) [13,14], isotope ratio analysis [15,16], lipidic profile [17,18] or a combination of these [11,19,20,21]. Excellent discrimination (100%) between wild and farmed Atlantic salmon was reported by Aursand et al. [13] by applying support vector machines (SVM) to data obtained by 13C NMR. In another study of the same group, the lipid extract was analyzed by 13C NMR and by gas chromatography with flame ionization detector GC-FID for fatty acid composition to discriminate between wild and farmed Atlantic salmon and assign the origin of the aquaculture samples to the farms included in the study [19]. The application of chemometrics to the reference farmed fish showed very good results for both approaches, but, surprisingly, slightly better for GC-FID data. The use of stable isotope analysis based on isotope ratio mass spectrometry (IRMS) is also a promising approach, especially when combined with chemical composition analysis, notably fatty acids [11,15,21]. Yet, previous works have demonstrated that lipidic profile is sufficient to establish the production method of salmon samples, particularly when combined with chemometric analysis [8,19,20]. Recently, Fiorino et al. [8] analyzed the lipid extracts obtained from a total of 100 samples of farmed and wild salmon by direct analysis in real time (DART) coupled to high resolution mass spectrometry (HRMS). The proposed methodology showed to be fast and allowed a good discrimination between the two groups (wild vs. farmed), though without differentiating the geographical origin of the farmed fish. Moreover, the referred approach requires advanced and expensive equipment, which is not available in most control quality/analytical laboratories. In the present study, the fatty acid composition of the same samples of wild and farmed salmon used in the work of Fiorino et al. [8] was analyzed by GC-FID, an affordable equipment commonly available in most laboratories. Subsequently, the obtained data were submitted to advanced chemometric analysis to establish the most suitable classifier able to discriminate the origin of salmon samples (wild vs. farmed, and the geographical origin among farmed samples) with the minimum possible computational effort.

## 2. Materials and Methods

### 2.1. Samples

In this study, a total of 100 authentic salmon samples obtained in the framework of the EU-funded project FOODINTEGRITY (Working Package 18) were analyzed. The samples included 26 wild salmon captured in Canada, and 74 farmed salmon samples from aquaculture farms in Canada (25), Norway (25) and Chile (24). No information was available about the gender of each specimen, neither of the diet or farming conditions used. The samples (entire fish) were transported frozen to the laboratory (Meriex Nutriscience, Chicago, IL, USA), allowed to defrost overnight at refrigerated temperature, and filleted in a cold room (4 °C). After removing the bones and skin, the muscles were grinded and distributed in labelled glass jars containing approximately 200 g each. The jars were immediately frozen and then shipped under freezing conditions (−20 °C) to the participating laboratories in different countries. After arriving, the samples were kept at −20 °C and submitted to lipid extraction as soon as possible.

### 2.2. Lipid Extraction

Lipids were extracted based on the Bligh and Dyer protocol [22] with some modifications. Briefly, about 13 g of each minced fillet were added with 13 mL of NaCl (1%) and 100 µL of butylated hydroxytoluene (BHT) (0.01% in n-hexane) to avoid oxidation, and homogenized for 1 min using an Ultra-Turrax at 13,500 rpm, keeping a low temperature by immersing the tube with the sample on ice. After that, 2.5 mL of the homogenate was transferred to a new tube and added with 2.5 mL of chloroform and 5 mL of methanol, both refrigerated. The solution was mixed vigorously by vortexing for 2 min. After centrifuging (4000 rpm, 15 min at 4 °C) the upper layer was discarded, and an additional 2.5 mL of refrigerated chloroform was added. After vortexing for 30 s and centrifuging under the same conditions, the chloroformic phase was transferred into a new tube and centrifuged (4000 rpm, 5 min at 4 °C). Finally, the chloroformic phase was collected into a previously weighted vial, flushed with a nitrogen stream and stored at −20 °C until further analysis. Each sample was submitted to independent extractions (*n* = 3).

### 2.3. Fatty Acids Analysis by GC-FID

Fatty acids were methylated using acid-catalysed trans-methylation with BF_3_ [23]. Firstly, the lipidic chloroformic extracts, previously stored at −20 °C, were dried under nitrogen and the tubes weighted to calculate the extraction yield. After dissolving the obtained lipids in 1 mL of n-hexane, for each sample, the volume containing 12.5 mg of lipids was transferred for a glass tube and dried under nitrogen. After adding 100 µL of BHT (0.01% in n-hexane) to prevent oxidation phenomena, fatty acid methyl esters were prepared. For that purpose, 1.25 mL of KOH (0.5 M) in methanol were added and the mixture was heated for 10 min at 100 °C after vortex-mixing vigorously. After cooling down the tubes, 1.0 mL of 14% boron-trifluoride in methanol (≥ 99.0% purity) (Sigma-Aldrich, Steinheim, Germany) was added to the solution, which was homogenized by vortexing, and the tubes heated again for 30 min at 100 °C. After completely cooling down the tubes in ice, 2.0 mL of n-hexane high performance liquid chromatography HPLC grade (Merck, Darmstadt, Germany) was added and the solution was vortex-mixed. Then, 1.0 mL of a saturated NaCl solution was added, followed by vigorous mixing and then by centrifuging for 5 min at 3000 rpm to obtain a clear upper phase. After that, 1.5 mL of supernatant was transferred to a new vial, added with anhydrous Na_2_SO_4_ and approximately 1.0 mL of FAME solution was transferred to an injection vial.

GC-FID analysis was carried out in a Shimadzu GC-2010 Plus gas chromatograph equipped with a Shimadzu AOC-20i auto-injector and a flame ionization detector (Shimadzu, Japan). FAME separation was achieved on a CP-Sil 88 silica capillary column (50 × 0.25 mm i.d., 0.20 μm, Varian, Middelburg, The Netherlands). The injector and detector temperatures were 250 °C and 270 °C, respectively. The oven parameters were set as follows: an initial temperature of 150 °C was increased at 3 °C/min to 160 °C and held for 2.0 min, then it was increased at 3 °C/min to 220 °C and held for 10 min. Helium was used as the carrier gas at a flow rate of 1 mL/min, and 1 μL of sample was injected using a split ratio of 1:50. Identification of compounds was performed by comparison of their retention times with those of authentic standards mixtures, namely 37 component FAME mix (certified reference material CRM47885) and PUFA nº.1 Marine source (standard 47,033) both from Supelco (Bellefonte, PA, USA). In addition, the fatty acid cis-11-octadecenoate (C18:1n7) was identified with and individual standard also purchased from Supelco. The results were expressed as the relative percentage of each fatty acid, calculated based on the chromatographic peak area. Each lipid extract was injected in duplicate.

### 2.4. Chemometric Analysis

#### 2.4.1. Dataset

The data used for chemometrics resulted from the chemical analyzes, totalizing 596 instances (4 chromatograms were excluded due to injection/chromatographic system problems) that were organized into four reasonably well balanced groups, each corresponding to a class of salmon: Norway Farmed (25 salmons), Chile Farmed (24 salmons), Canada Farmed (25 salmons), Canada Wild (26 salmons). Each salmon sample was represented by a block of 6 chromatograms. The number of independent features considered was 17, corresponding to the identified fatty acids.

#### 2.4.2. Statistical Analysis by One-Way ANOVA

The differences between groups were analysed using a one-way analysis of variance (ANOVA) followed by Tukey’s honest significant difference post hoc test with *p* = 0.05. The analysis was carried out using the SPSS v. 23.0 program (SPSS v. 23.0; IBM Corp., Armonk, NY, USA).

#### 2.4.3. Data Modelling Tools

The data modelling tools used in this work are based on the Orange 3.24 software, which, in turn, uses libraries from the Scikit-learn, Numpy and Scipy written in Python. The graphical user interface uses the cross-platform Qt framework.

##### Data Visualization by PCA and t-SNE

As a first approach, the possibility of separating the data by classical and linear statistical methods was evaluated. For that purpose, principal component analysis (PCA) was used to check the possibility of obtaining a separation by linear composition in a subspace of principal components based on the PCA projections. When the PCA shows data superposition among groups, it means that the possibility of separating groups in the original dimension space cannot be performed, since the mapping from the original dimension space to the principal component space is always linear [24]. A manner to overcome this issue involves using the t-distributed stochastic neighbor embedding (t-SNE) method, which is able to replicate non-linear mappings in the original data space to the lower dimension [25]. Thus, a non-linear approach by t-SNE was used to observe separations in higher dimensions when they are projected in a two-dimensional space.

##### Machine Learning Classifiers

Several well-known classification models were evaluated, namely k-nearest neighbors (kNN), decision tree, support vector machine (SVM), random forest, artificial neural networks, naïve Bayes and AdaBoost, whose main characteristics are described as follows:

- kNN is a method that can be used for data classification. A sample is classified by the rating vote of its neighbors present in the dataset; the result is attributed to the most common class among the k closest neighbors. If k = 1, the object is simply assigned to the class of the only nearest neighbour [26].
- Decision tree is one of the predictive modeling approaches used in machine learning. It uses a tree schema as a predictive model to move from the analysis of a sample to conclude the class that corresponds to the sample. The decision trees are built inductively from a dataset that is analyzed by measures, such as the information entropy. During the process, the dataset is divided, successively, in order to reduce the uncertainty in the classification. That division is represented by each feature (tree node) [27].
- Support-vector machines (SVM) are models of supervised learning. Given a set of training examples, each is marked as belonging to one or the other of two categories. The resulting model, after training, is a representation of the examples as points in space, mapped so that the widest possible spatial margin separates the examples of the two categories. The new examples are expected to belong to a category based on the margin side where they are located. That side is formed from the center of the separating margin. This process leads to the fact that SVM can generalize as best as possible, avoiding overfitting. In addition to performing linear mapping, SVM can efficiently perform non-linear mapping using what is called a kernel trick, implicitly mapping their inputs into high-dimensional spaces, allowing separations to happen in the high dimensional space [28].
- Random forests are a learning method that can be used for classification. They are built based on decision trees, but in this case, there is a participation of several trees that are trained with segments of the dataset and with segments of the feature set randomly selected [29]. This stochastic factor improves the generalization of the model and reduces the overfitting.
- Artificial neural networks (ANN) is a model based on a collection of connected units or nodes called “artificial neurons”, which mimic neurons in a biological brain. Each connection can transmit signals from one artificial neuron to another; the magnitude of the signal is modulated by a parameter adjusted during the learning phase. Each neuron behaves like a separating hyperplane in the classification space. The association of neurons, by layers, allows obtaining conjugations of complex hyperplanes that lead to non-linear classification models. The adjustment of the parameters is made by algorithms that use the gradient descent of the error. The error is defined by the difference between the value emitted by the neural network and the desired value [30].
- Naïve Bayes, in machine learning, are probabilistic classifiers, based on the application of Bayes’ theorem with evidence on the assumptions of independence between features [31]. Naïve Bayes classifiers are easy to implement using Gaussian curves and the inverse Bayes formula.
- AdaBoost (short for adaptive boosting) is based on the idea that a set of weak classifiers can result in a strong classifier. Weak classifiers are combined linearly, but modulated by coefficients that are obtained during the training. The choice of weak classifiers is made focusing on the examples that are classified with more difficulty. In this iterative process, the weak classifiers have coefficients that correspond to the classifier error on the dataset. The weak classifiers that make the least mistakes have their coefficients increased. The strong classifier aggregates all those weak classifiers according to their importance coefficients [32].

All these models are mappers with non-linear capabilities, each having different methods of statistical induction of knowledge. Thus, some may perform better for certain classification problems than others. For this reason, in this study we used a test bench formed by several models.

All these classifiers were developed/trained, in a first phase, using the 17 features present in the dataset and the obtained classification results used for the assessment of each model.

##### Reduction in Features

Aiming at decreasing the cost of analyses and complexity of data, accelerating the whole process, it is frequently important to reduce the number of features in chemometric analysis. On the other hand, the reduction in the number of features can enhance the generalization capability of the classifier. Therefore, after having the models parameterized using all the features, strategies were developed to explore the reduction in features. The selection of the best features was made using a ranking process that is based on the measurement of information entropy. In this case, the well-known information gain ratio criterion was applied [27]. This criterion measures the uncertainty in how the data are separated based on a specific feature; the value of the information gain ratio is calculated for each feature, representing its separation power in the dataset. The sorting of features, according to these values, establishes their ranking. This criterion is normalized regarding the number of data partitions that the usage of a given feature causes. This mechanism makes it possible to obtain a numerical criterion, independent from the classifying bias (overfitting), prompted by numerous potential partitions of information groups. Thus, in the next step, the minimum number of the best features, in that ranking, was determined, ensuring that the classification model still classifies the data accurately.

Aiming to evaluate model overfitting, assertiveness and generalization assessments of the classification models were made using both external and full-cross validation. For external validation, the test dataset was obtained by splitting the data into 20:80. For the cross-validation scheme a mechanism of leave-one-sample-out (each sample corresponding to a block of 6 chromatograms) was used. Moreover, that scheme allowed us to parameterize the models by observing the validation performance given by the average of all six-chromatogram groups. The used performance indicators were the accuracy (CA) and F1. F1 is a more revealing measure of the practical performance that a classification model presents, being more sensitive to poorly classified instances. Moreover, an assertiveness analysis was made by using confusion matrices.

During the process of feature reduction, the performance of the classifiers was tested using a successive bisection approach. Starting from a set of classification models that normally provide high assertiveness rates and using all the features sorted by the information gain ratio, the following method (Algorithm 1) was developed and applied. This algorithm allows for the optimization of the search for the minimum number of features to classify the samples, with an arbitrary minimum of 99% of accuracy.
**Algorithm 1** Searching the optimal number of featuresGiven a set of features *F* of *n* elements with gain ratio values *F*_0_, *F*_1_, *F*_2_, …, *F_n−1_* sorted such that *F*_0_ > *F*_1_ > *F*_2_ … > *F_n−1_*, and the *accuracy_m_* being the correctness classifying the dataset using the first *m* features. The following algorithm is based on the binary search to find the index *m* in *F* that corresponds to the minimum index to classify the dataset properly.Set *L* to 0 and *R* to *n* − 1Set *m* = *R*, *m_old_* = *m*If *accuracy_m_* < 99%, stop, the classifier must use the all featuresSet *m* (the middle position) to the *floor* of $\frac{L+R}{2}$If *accuracy_m_* > 99%, set *R* to *m*If *accuracy_m_* < 99%, set *L* to *m*If abs(*m* − *m_old_*) > 0, *m_old_* = *m*, goto 4Stop, the classifier must use the first *m* + 1 features

The algorithm is repeated independently for each of the classifiers under analysis. The minimum number of features is selected to further actions when the accuracy is ≥ 99% for at least one model. For each machine learning classifier, a trial-and-error approach was used to find the best parametrization, with classifiers being tuned at two stages. At the first stage, Algorithm 1 was applied to all the models using the maximum number of features (seventeen). This tuning aims at obtaining a good classification, concerning the dataset, for each classifier. The adjustment was done manually, in a trial-and-error fashion, changing the hyperparameters associated to each model. In this phase, to get a good functional response (selection) from Algorithm 1, it is not necessary to have a perfect tuning of the classifiers.

**Figure 1:**
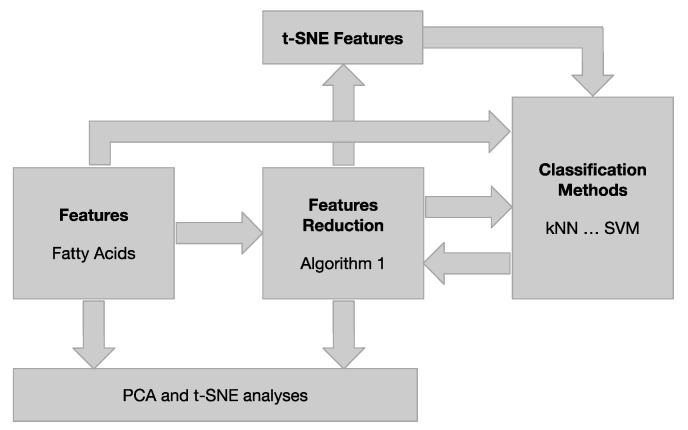
Main process pipeline. PCA: principal components analysis; t-SNE: t-distributed stochastic neighbor embedding.

After this, the minimum number of features required to produce good classifications (accuracy of 99%) on a classifier are known. Thus, at the second stage, eventually, one could make new adjustments to the classifier models to improve functional performance subjected to the new subset of features selected after applying Algorithm 1. The details of the final parameters used for the best models are shown in Table 1. Figure 1 schematically describes the chemometric approaches and main process pipeline used in this work.

## 3. Results and Discussion

### 3.1. Fatty Acids Composition

**Figure 2:**
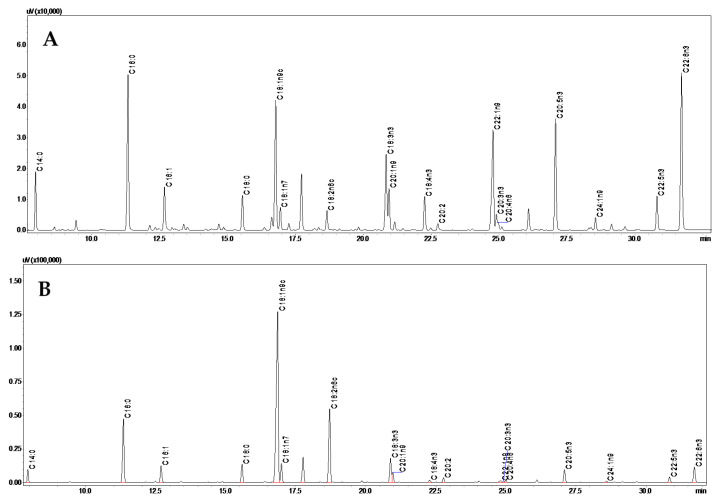
Chromatograms of fatty acid profiles obtained by GC-FID analysis of wild (**A**) and farmed (**B**) salmon samples from Canada.

Figure 2 shows representative chromatograms of fatty acid analysis obtained from wild and farmed salmon samples and Table 2 presents their relative contents for the four salmon groups under evaluation, namely, wild from Canada and farmed from Canada, Chile and Norway.

Striking differences can be observed between wild and farmed salmons, namely in terms of the sum of MUFA and PUFA, ratio between omega-3 and omega-6 fatty acids, and also regarding several individual fatty acids. For the same amount of derivatized lipids, and when compared to wild, farmed salmon presented a significantly higher (*p* < 0.05) content of oleic and linoleic acids and lower contents of EPA, DHA and C22:1 isomers. In general, the obtained results are in good agreement with previous knowledge since farmed salmons are frequently described as having higher amounts of C18:1, C18:2 and C18:3 fatty acids, while wild are richer in long chain omega-3 PUFA as well as saturated fatty acids (SFA) [6,7]. Nevertheless, in the present study, similar contents of α-linolenic acid (C18:3n3) were found between the wild and farmed groups and only a slightly higher amount was verified in terms of SFA. The obtained data confirm that the consumption of wild salmon can be associated with greater health benefits due to their favorable ratio omega-3/omega-6 fatty acids. As discussed in previous papers, the differences observed are most probably related with differences in the diets of fish from the wild and in aquaculture conditions [6,17,21].

Compared to the results previously reported for the analysis of the same samples (as part of the EU-funded project FOODINTEGRITY) using a different technique, namely DART-MS, some quantitative differences can be pointed out. Namely, the content reported by Fiorino et al. [8] for 16:0 was higher in both farmed and wild groups, while the present GC-FID results show higher contents for 18:3, 18:1 (mainly for the farmed group) and 22:6 (mainly for the wild group). These dissimilarities can be due to the different techniques used, one based on mass spectrometry and normalized abundances, and the other relying on flame ionization detection and relative peak areas.

### 3.2. Chemometric Analysis of the Generated Data

#### 3.2.1. Features Selection

The importance of each feature regarding the group separation was evaluated by applying the information gain ratio criterion, as described in the materials and methods section. Table 3 presents the ranking of features obtained based on that measurement. Subsequently, the developed algorithm (Algorithm 1) was used to determine the minimum number of features required for classifying the four groups accurately. That number was found to be six, corresponding to the following features: 16:0, 18:2n6c, 20:3n3 + 20:4n6, 14:0, 18:1n9 and 22:6n3.

#### 3.2.2. Data Visualization by PCA and t-SNE

**Figure 3:**
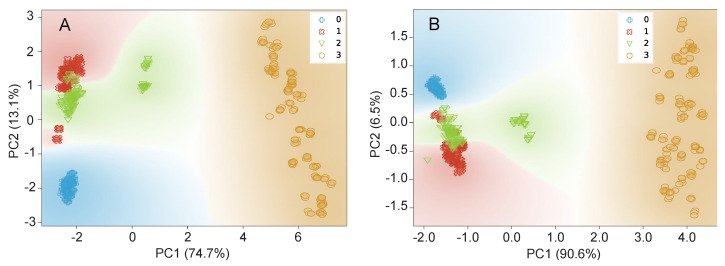
Scatterplot obtained for the first two principal components after applying PCA to the whole dataset using (**A**): all the 17 features, (**B**): the 6 best features (16:0, 18:2n6c, 20:3n3 + 20:4n6, 14:0, 18:1n9 and 22:6n3); 0—Norway farmed, 1—Chile farmed, 2—Canada farmed, 3—Canada wild.

As a first approach, PCA was applied to the dataset as a linear and unsupervised statistical method. This method is one of the most widespread exploratory data analysis tools, providing a fast data overview by projecting each data point onto a small number of principal components, thus reducing data dimensionality, while maintaining their variation as much as possible [24]. Moreover, this approach was used previously regarding the analysis of the same salmon samples by a distinct methodology, namely DART-MS analysis [8]. Figure 3A presents the data distribution on two principal components when all the 17 data features are used. As it can be observed, PC1 and PC2 accounted for 87.8% of the total variance and showed a clear separation between the wild samples and the farmed ones, similarly to the results reported by Fiorino et al. [8]. Although it was not possible to clearly distinguish the farmed samples according to their geographical origin, mainly due to overlapping of samples from farmed Canada and Chile groups, a better separation was achieved when compared to the results of Fiorino et al. [8] using DART-MS analyses. Interestingly, in their work, five out of the six fatty acids, exhibiting the most relevant differences between wild and farmed salmons, were in common with the ones selected by Algorithm 1. Linolenic acid (C18:3) was an exception because in the present work it ranked as the 15th position with a low information gain ratio value, thus not being relevant to distinguish the four groups using the GC-FID fatty acid profiles. Subsequently, PCA was also applied to the whole dataset, but using only the selected best six features (Figure 3B), evidencing results similar to the ones obtained with all the 17 features.

**Figure 4:**
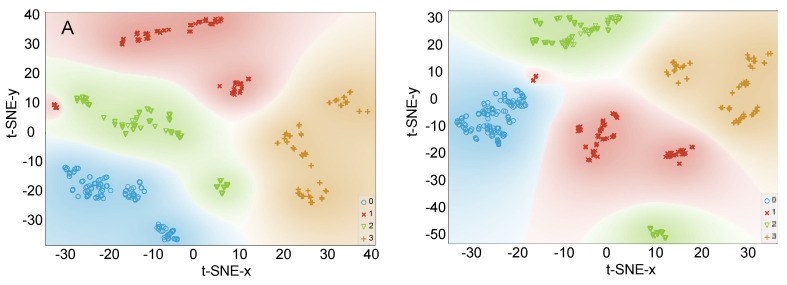
Scatterplot obtained after applying t-SNE to the whole dataset using (**A**): all the 17 features (**B**): only the 6 best features (16:0, 18:2n6c, 20:3n3 + 20:4n6, 14:0, 18:1n9 and 22:6n3); 0—Norway, Figure 1. Chile farmed, 2—Canada farmed, 3—Canada wild.

The interpretation of Figure 3A,B allows drawing two conclusions: (1) most of the data are strongly explained by the first principal component regardless of the number of used features, namely all the 17 or only the best six, which confirms that most of the features are not important for the correct classification; (2) some samples of Chile farmed and Canada farmed groups are not linearly separable with data projected on a 2D subspace, thus suggesting the need for non-linear classification models. Therefore, t-SNE was applied to the dataset, first using all the 17 features, and then only the selected best six (Figure 4A,B). This method allows the projection of the original dimension on two dimensions without losing the non-linear relations presented at the high dimensional space. Thus, it is a suitable tool to perceive the separability of groups at the original dimension. As shown in Figure 4, there is no data superposition and, in general, the groups are well separated according to this method. This information suggests a good data separability when the classification models can handle non-linearities in a high dimension space.

A good separability among groups was also observed when the number of employed input features was only six (Figure 4B). This suggests that, in the high dimension original space, the separability is achieved based on only a few features. Normally, this is an advantage for subsequently used classifiers because it promotes generalization and tends to avoid overfitting, thus strongly suggesting that new samples will be properly classified based on such classifiers.

#### 3.2.3. Machine Learning Classifiers

In this work, a total of seven different classifiers were tested considering performance (classification accuracy) and required computational effort (evaluated as test time). Similarly, as was done for PCA and t-SNE, each classifier was first assayed using all the 17 features as inputs to the classifiers. The obtained performance is shown in Table 4, evidencing that ANN, random forest, SVM, naïve Bayes and kNN were the best models as they showed a maximum performance, allowing classifying, without error, for all of the test dataset. Nevertheless, they are closely followed by the remaining classifiers, with decision tree being the one that performed worst. In terms of performance time (test time), among the classifiers that allowed 100% accuracy (CA), naïve Bayes was the best one. This can be explained by two factors: first, one must consider that in this case the number of features exceeds the needs, thus, according to Occam’s razor principle, the simpler model can achieve a good performance; second, as the model is very simple to implement, the number of required computational calculation steps is small, thus corresponding to a shorter time of performance.

Next, the performance of classifiers was assayed with only the six best features as their inputs. As can be observed in Table 5, in this case, the ANN, SVM and kNN classifiers allowed 100% correct classification, as measured by accuracy and F1 indicators. It is possible that the elements that were not correctly classified by the remaining models do not have statistical significance to change the parameters present on the learning mechanism to the rest of classifiers. Among the best classifiers the one that presented the best computational performance was the SVM.

Overall, the remaining classifiers were very close to the performance of ANN, SVM and kNN, despite the reduced number of features used. For this reason, it was decided to further observe the classification performance when the features are remapped by the t-SNE method as inputs for the classifiers, keeping the same parametrization for all models, as in the previous scheme. By applying Algorithm 1 and extending the processing pipeline with the t-SNE block, namely by placing that block between the features used and the classifiers, it was possible to conclude that the classification can still be performed successfully by relying on only three features, namely 16:0, 18:2n6c and the sum of 20:3n3 + 20:4n6. The obtained results are presented in Table 6, evidencing 100% accuracy of sample classification using the kNN, with only three compounds being required in this model. In this scenario, the decision tree classifier showed the worst performance, being the only one presenting an accuracy < 95%.

**Figure 5:**
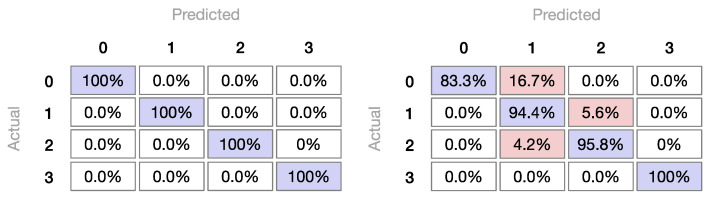
Confusion matrix (showing proportion of actual) for the decision tree model (**left**) and confusion matrix for the kNN model (**right**), both processing only three features. 0—Norway farmed, 1—Chile farmed, 2—Canada farmed, 3—Canada wild.

Figure 5 shows the confusion matrices, evidencing sample classification, for the best (kNN) and worst (decision tree) models, using only the three best features, as processed by t-SNE. While the confusion matrix for the kNN model presents all samples as being correctly classified, the confusion matrix for the decision tree evidences some errors because six samples from group zero (Norway farmed) were misclassified as being from group one (Chile farmed). This shows that the inductive learning mechanism present in the decision tree was not able to classify those samples correctly, as probably happens with the remaining classifiers, except for kNN that is not based on inductive learning.

## 4. Conclusions

In general, the four evaluated groups of salmon (wild from Canada and farmed from Canada, Chile and Norway) showed different fatty acid profiles, with wild specimens presenting significantly higher contents of health beneficial omega-3 fatty acids, in particular DHA and EPA, while farmed salmon presented significantly higher (*p* < 0.05) amounts of oleic and linoleic acids. Among the three groups of farmed salmon with different geographical origins, specimens from Chile and Canada were more similar, with the ones from Norway being more distinct mainly due to their lower levels of SFA and higher levels of α-linolenic acid. The differences among farmed groups are most probably related to different types of feed used in each farm. However, information about relevant factors such as farming diet and conditions, which are known to affect the lipidic composition of fish, was not available. In this work, we demonstrated the possibility of discriminating between wild and farmed salmons, as well as differentiating the origin within farmed ones, based on the use of machine learning models applied to fatty acid composition obtained by GC-FID. Thus, compared to a previous approach reported for the same samples, namely the use of PCA applied to normalized intensities of the most abundant signals generated by DART-HRMS analysis of the lipid extracts, this method showed a higher discrimination power. Moreover, this method proved to be simple and it only requires the use of affordable equipment, commonly found in most laboratories. Nevertheless, this approach has the disadvantage of requiring a longer analysis time compared to DART-HRMS. The developed algorithm combined with the information gain ratio criterion allowed us to establish the number of optimal features, so the classification tasks can still attain a very good performance. The feature reduction offers a computational speedup during the classification process. Among the seven tested machine learning models, the best results were obtained with the k-nearest neighbors (kNN) classifier, allowing for the correct classification of all tested samples. Moreover, it was shown that using t-SNE in the processing pipeline boosts the reduction in features, while still maintaining 100% accuracy in data classification. The performance difference between the test dataset and the leave-one-sample-out cross-validation was residual, meaning a good generalization figure.

## Figures and Tables

**Table 1:** Details of the parametrization used to tune each of the final classification models.

Classifier	Parameters
kNN	Number of neighbors: 3; Metric: Euclidean; Weight: Distance.
Decision tree	Limit the tree depth: 100; Do not split subsets smaller than: 2; Min. number of instances in leaves 3.
SVM	C: 15; Kernel: Radial Basis Function (RBF); g: auto.
Random forests	Number of trees: 15; Do not split subsets smaller than: 5.
ANN	Neurons in hidden layers: 300; activation: Rectified Linear Unit (Relu); solver: Adam; regularization: 0.02.
Naive Bayes	Non-applicable
AdaBoost	Number of estimators: 80; learning rate: 0,7; classification algorithm: SAMME.R; Regression loss function: Square.

kNN: k-nearest neighbors; SVM: support vector machine; ANN: artificial neural networks.

**Table 2:** Fatty acid composition (relative% of the identified FAME) obtained by GC-FID analysis of lipids from the wild and farmed salmon samples of different origins. Results are given as mean ± SD of the total specimens analyzed for each group.

Fatty Acid	Wild	Farmed		
	Canada (*n* = 26)	Canada (*n* = 25)	Chile (*n* = 24)	Norway (*n* = 25)
14:0	3.86 ± 0.40 ^c^	1.99 ± 0.47 ^a^	1.98 ± 0.10 ^a^	2.14 ± 0.06 ^b^
16:0	15.75 ± 1.70 ^d^	11.64 ± 0.83 ^b^	12.22 ± 0.78 ^c^	9.13 ± 0.24 ^a^
16:1	3.73 ± 0.58 ^c^	3.48 ± 0.80 ^b^	2.78 ± 0.25 ^a^	2.67 ± 0.07 ^a^
18:0	3.61 ± 0.65 ^b^	3.54 ± 0.22 ^b^	3.78 ± 0.31 c	2.60 ± 0.10 ^a^
18:1n9c	12.89 ± 2.89 ^a^	42.15 ± 4.42 ^b^	41.98 ± 1.58 ^b^	43.89 ± 0.29 ^c^
18:1n7	2.19 ± 0.28 ^a^	3.82 ± 0.33 ^b^	4.05 ± 0.41 ^c^	3.81 ± 0.28 ^b^
18:2n6c	1.76 ± 0.15 ^a^	14.35 ± 1.52 ^b^	16.36 ± 0.72 ^c^	16.31 ± 0.21 ^c^
18:3n3	6.72 ± 1.52 ^c^	5.02 ± 0.57 ^b^	4.47 ± 0.40 ^a^	7.79 ± 0.27 ^d^
20:1n9	3.57 ± 0.94 ^c^	2.09 ± 0.65 ^a^	2.36 ± 0.22 ^b^	2.17 ± 0.24 ^a^
18:4n3	2.51 ± 0.51 ^c^	0.58 ± 0.14 ^a^	0.61 ± 0.09 ^a,b^	0.69 ± 0.08 ^b^
20:2n6	0.48 ± 0.10 ^a^	0.87 ± 0.09 ^b^	1.04 ± 0.06 ^c^	0.90 ± 0.06 ^b^
22:1n11 + 22:1n9	9.30 ± 2.90 ^b^	1.10 ± 1.11 ^a^	0.64 ± 0.27 ^a^	0.67 ± 0.05 ^a^
20:3n3 + 20:4n6	1.83 ± 0.33 ^d^	0.79 ± 0.11 ^c^	0.60 ± 0.05 ^a^	0.66 ± 0.02 ^b^
20:5n3	9.12 ± 0.97 ^c^	2.74 ± 0.42 ^b^	2.54 ± 0.23 ^a^	2.69 ± 0.15 ^ab^
24:1n9	1.06 ± 0.17 ^c^	0.24 ± 0.05 ^b^	0.21 ± 0.02 ^a^	0.26 ± 0.02 ^b^
22:5n3	2.75 ± 0.30 ^d^	1.37 ± 0.34 ^c^	1.28 ± 0.13 ^b^	1.17 ± 0.05 ^a^
22:6n3	18.83 ± 2.82 ^d^	4.23 ± 0.62 ^c^	3.12 ± 1.11 ^b^	2.45 ± 0.13 ^a^
Σ SFA	23.25 ± 1.93 ^d^	17.18 ± 1.42 ^b^	17.98 ± 1.13 ^c^	13.6 ± 1.98 ^a^
Σ MUFA	32.73 ± 3.22 ^a^	52.89 ± 1.76 ^c^	52.02 ± 1.63 ^b^	52.41 ± 7.55 ^c^
Σ PUFA	43.95 ± 2.77 ^c^	29.93 ± 0.62 ^a^	30.00 ± 1.62 ^a^	31.99 ± 4.62 ^b^
n3/n6	16.75 ± 1.61 ^b^	0.90 ± 0.24 ^a^	0.66 ± 0.04 ^a^	0.82 ± 0.02 ^a^

SFA: saturated fatty acids; MUFA: monounsaturated fatty acids; PUFA: polyunsaturated fatty acids. Different letters indicate significant differences (*p* < 0.05) between groups in the statistical analysis by one-way analysis of variance (ANOVA).

**Table 3:** Features sorted by applying the information gain ratio criterion.

Fatty Acid	Gain Ratio
16:0	0.719
18:2n6c	0.709
20:3n3 + 20:4n6	0.675
14:0	0.615
18:1n9c	0.562
22:6n3	0.548
20:2n6	0.523
22:1n11 + 22:1n9	0.506
24:1n9	0.505
22:5n3	0.464
18:1n7	0.461
18:4n3	0.446
20:5n3	0.423
16:1	0.402
18:3n3	0.378
20:1n9	0.366
18:0	0.353

**Table 4:** Classifiers performance, in the test dataset, using all the 17 input features.

Model	Test Time (s)	CA	F1
ANN	0.011	1.000	1.000
Naïve Bayes	0.008	1.000	1.000
kNN	0.012	1.000	1.000
SVM	0.013	1.000	1.000
Random Forest	0.011	1.000	1.000
AdaBoost	0.0064	0.991	0.991
Decision Tree	0.001	0.908	0.908

CA: accuracy; F1 score: harmonic mean of the precision and recall.

**Table 5:** Classifier performance, in the test dataset, using the selected best 6 input features.

Model	Test Time (s)	CA	F1
ANN	0.010	1.000	1.000
SVM	0.006	1.000	1.000
kNN	0.009	1.000	1.000
Random Forest	0.011	0.992	0.992
Naïve Bayes	0.003	0.992	0.992
AdaBoost	0.004	0.983	0.983
Decision Tree	0.001	0.983	0.983

**Table 6:** Classifiers performance, in the test dataset, using the selected best 3 input features mapped by t-SNE.

Model	Test Time [s]	CA	F1
kNN	0.094	1.000	1.000
SVM	0.016	0.992	0.992
Random Forest	0.021	0.992	0.992
ANN	0.112	0.983	0.983
AdaBoost	0.020	0.967	0.967
Naïve Bayes	0.018	0.967	0.967
Decision Tree	0.001	0.925	0.925
